# Developing and validating a tool for assessing the confidence in the competence of midwifery tutors in India on WHO core competency domains

**DOI:** 10.1371/journal.pgph.0003626

**Published:** 2024-08-29

**Authors:** Paridhi Jha, Bharati Sharma, Prabhu Ponnusamy, Purna Chandra Sahoo, Vikas Kumar Jha, Nishtha Kathuria, Devika Mehra, Sunanda Gupta, Arvind Pandey, Ram Chahar, Frances Emma McConville, Medha Gandhi, Malin Bogren

**Affiliations:** 1 Foundation for Research in Health Systems (FRHS), Bengaluru, Karnataka, India; 2 Indian Institute of Public Health, Gandhinagar, Gujarat, India; 3 Mamta Health Institute of Mother and Child, New Delhi, India; 4 World Health Organization, India Office, New Delhi, India; 5 World Health Organization, Headquarters, Geneva, Switzerland; 6 Bill and Melinda Gates Foundation, Lead, Program Advocacy: Family Health, Immunization & Infectious Diseases, New Delhi, India; 7 Institute of Health and Care Sciences, University of Gothenburg, Gothenburg, Sweden; PLOS: Public Library of Science, UNITED STATES OF AMERICA

## Abstract

Negligible quantitative research evidence exists on standardisation and psychometric validation of questionnaires that measure midwifery educators’ confidence in their competence. This study developed a self-assessment of confidence in competence questionnaire in India based on the WHO Midwifery Educator Core Competencies (2014) with an aim to develop and validate a self-assessment tool measuring midwifery tutors’ confidence in competence in imparting quality midwifery education. The questionnaire was developed as part of a multi-centre study to identify confident midwifery tutors for further training as educators, supporting India’s rollout of professional midwives. The questionnaire underwent rigorous psychometric testing among 2016 midwifery tutors in India. Following exploratory Principal Component Analyses (PCA), the nine core competencies outlined in the WHO document were analysed separately. The results indicate that the questionnaire is psychometrically valid, with an internal consistency range of 0.81–0.93 for the nine domains. This robust testing process ensures the reliability and validity of the questionnaire. The self-assessment questionnaire can potentially be a valuable tool in India and other high-, middle-, and low-income countries. From a programmatic perspective, it can help identify key gaps and prioritise training needs, particularly in low-resource settings, so that limited resources are best utilised to fill the most prominent gaps. Furthermore, it can provide a universal platform for comparing data from different settings, facilitating global collaboration and learning in midwifery education.

## Introduction

A competent health workforce–including professional midwives–is known to improve the performance of health systems, ensuring that the healthcare is evidence-based, skilled, sensitive, integrated, and person-centred [1]. Global evidence shows that professional midwives whose education has been informed by global standards [2] are the most effective and economical means of promoting safe healthcare for women of all age groups and their neonates [3–5]. However, competent midwifery educators are an essential component in preparing competent midwives [6]. Competent midwifery educators are capable of training their students to meet the global education standards set by the International Confederation of Midwives (ICM) and the World Health Organization (WHO) [7, 8]. Thus, there is an urgent need to make investments in building midwifery educators’ competence in teaching midwifery, especially in low- and middle-income countries (LMICs) [9]. A baseline measure of midwifery educators’ competency is essential before initiating improvement measures to bridge the identified gaps. Usually, long-term assessments and direct observations for accurate measures of competence have been recommended for licensure [10, 11]. However, they may prove too costly and time-consuming to track the ongoing status of competencies [12]. Furthermore, measuring competence in its truest form for any profession has been reported as challenging due to its multi-dimensional nature [13] and close association with confidence and performance.

### Competence, confidence, and performance

Concepts of confidence and competence are not synonymous. The Oxford Dictionary defines *confidence* as a feeling of self-assurance from an appreciation of one’s abilities or qualities, while *competence* is ’the ability to do something successfully or efficiently.’ Confidence has three influencing attributes: situational, institutional (structure and pedagogy of educational programmes), and dispositional (personal traits- attitudes and motivation) [14–17].

When applied to the midwife, confidence means task performance and perseverance when confronting difficulties and setbacks in work situations. Butler et al. identified ’being a safe practitioner’ as one of the essential competencies required of a midwife at the point of registration [18], the ability to detect deviations, take appropriate action, and respond to emergency situations [19]. In fact, when viewed from a performance orientation, competence reflects situational relationships among providers, their patients, and the systems in which they perform and, thus, is only partly dependent on the attributes of individual actors [20]. One can say confidence is "situated" competence [21].

Therefore, confidence seems to be a mediating factor for achieving and demonstrating competence and partially predicting performance. For the proposed study, measuring the confidence of midwifery educators could partly reflect the competence of midwifery tutors in India in providing quality teaching-learning experiences to their students.

Several cross-sectional research studies have used the respondents’ expressed confidence as a reliable indicator of their self-assessed competence [22–25]. Therefore, midwives with less confidence might be unable to perform acceptable clinical practice [26]. It would be reasonable to hypothesise that the same may apply to midwifery educators.

Some studies have analysed strategies to build midwifery educators’ competence [27–29], and a recent study reported the findings on midwives’ competence using a validated tool [30]. However, no tool–to the best of the authors’ knowledge–measures the competence of midwifery educators against global standards [2.8]. Some existing tools cover the quality of midwifery education [31, 32]. However, none seem to measure the educators’ confidence or competence in organising and imparting quality education. Global standards and indicators for quality midwifery education may not bring about any improvements unless the educators are confident in their ability to organise learning sessions and teach using appropriate pedagogy.

### Background of midwifery education in India

In a landmark move, the Government of India (GoI) launched the Midwifery Service Guidelines [33] to reduce maternal and neonatal morbidity and mortality. As such, India must educate 90,000 midwives to cover all round-the-clock public health facilities offering childbirth services within the next 3–5 years [34].

The Guidance document [34] outlines the process of establishing several National Midwifery Training Institutes (NMTIs), which would support the State Midwifery Training Institutes (SMTIs) in educating professional midwives. Under the initiative, the midwifery educators will first be trained and deployed to the NMTIs and SMTIs [35] and then start educating the professional midwives—termed Nurse Practitioners in Midwifery (NPMs)—in India.

In light of this development, having a tool that measures the confidence in the competence of midwifery tutors would serve well as a programmatic tool to identify midwifery tutors from current integrated midwifery programmes who already demonstrate better confidence. There was a need to identify the baseline competencies of available tutors in India who teach midwifery against a global standard so that improvements during midwifery educators’ training were driven by evidence. Therefore, this study aimed to develop and validate a self-assessment tool measuring midwifery tutors’ confidence in competence in imparting quality midwifery education.

## Methodology

### Development of the questionnaire

The WHO Core Competencies of Midwifery Educators [2] was the primary document to develop the competence statements. Each core competency was coded to identify key competencies it covered and was broken down into competence statements (hereafter termed as items) so that each item specifically explored the respondents’ confidence in one competence. This meant a single competency in the WHO document could be broken into multiple items, holistically covering said competency.

Thereafter, three co-authors carefully read all recently published literature [10, 29, 36–39] independently and discussed their reflections to identify if any relevant item on confidence could be added. The review stopped when no new items could be identified, and no new competency studies post-dating the WHO Core Competencies of Midwifery Educators could be found. In total, 119 items were formulated (Table 1), covering the nine WHO core competencies of midwifery educators [2].

**Table 1:** Number of items drafted under each WHO core competency.

Core Competency		Number of items drafted
Competency 1	Midwifery educators create an environment that facilitates learning	28 items
Competency 2	Midwifery educators create an environment for effective teaching of clinical practice of midwifery	21 items
Competency 3	Midwifery educators conduct regular monitoring, evaluation and assessment of programmes and students	12 items
Competency 4	Midwifery educators maintain current knowledge and skills in midwifery theory and practice in accordance with best available evidence	15 items
Competency 5	Midwifery educators participate in formulating policies and programme outcomes and in designing and implementing curricula	11 items
Competency 6	Midwifery educators are effective communicators and function as advocates, change agents and leaders	15 items
Competency 7	Midwifery educators incorporate and promote ethical aspects of midwifery care in teaching/learning activities by consistent role modelling	7 items
Competency 8	Midwifery educators incorporate and promote legal aspects of midwifery care in teaching/learning activities by consistent role modelling	5 items
Competency 9	Midwifery educators promote the use of research and use it to inform midwifery education and practice	5 items
	Total	119 items

Each item prompted the participant to rate their confidence in performing that competence on a 5-point Likert scale (1: Not at all confident; 5: Very confident). A group of senior midwifery experts (n = 17) from India reviewed the draft self-assessment questionnaire. The senior midwifery experts were selected based on: 1) their experience of teaching midwifery in integrated courses, 2) representing both the public and private sector, 3) being a member of curriculum committees/ academic committees, and/or 4) serving as a principal and having the administrative experience of making a midwifery education programme functional in Indian setup.

Based on their suggestion, the top three competencies in the WHO document (legal and ethical aspects and research) were moved to the very end of the questionnaire. The senior midwifery experts judged these to be more complex concepts that could potentially de-motivate the respondents, affecting the completion rates on filled questionnaires.

Furthermore, the midwifery experts opined that the socio-cultural and legal context of midwifery education in any country greatly impacted the scope of practice of midwifery tutors. Therefore, there could be a few items in the draft questionnaire that the tutors could not answer on a Likert scale because the competence covered in such items was not part of their legal scope of practice (eg. having the authority to practice hands-on midwifery care after becoming a tutor, as the dual role of practising midwife and midwifery tutor was not mandated by either the Indian Nursing Council or any other Government guidelines). Therefore, low confidence could have underlying issues beyond an individual tutor’s confidence in competence.

To improve the questionnaire’s fairness (so that the respondents were not forced to choose “not confident” without sharing the reason/s behind it), sets of contextual questions were added before all nine WHO core competencies, where applicable, based on the input of the midwifery experts. After deliberation, co-authors decided not to add the contextual questions as part of the confidence in competence questionnaire because these contextual questions were more to assess the situation in which the tutors were teaching midwifery rather than exploring their confidence in competence.

#### Step 1: Confirming the content validity index (CVI) of questionnaire items.

Beck and Polit define content validity as the degree to which an instrument has an appropriate sample of items for the measured construct [36]. It is an important procedure in scale development. The content validity index (CVI) is the most widely used index, and it is calculated by presenting a new draft scale to a group of experts (n = 2–8) to seek their input on how relevant they find each item for the measured construct [37]. The responses are usually collected on a three- or five-point Likert scale. for the 5–7 experts’ panel, a CVI of 0.83 is considered acceptable. Any item with a CVI of 0.83 to 1 should be retained as is. Items with CVI 0.6–0.82 should be reframed and CVI should be checked again. Items with CVI <0.6 should be discarded.

The current scale was presented to seven Indian midwifery experts (Nurse-midwives who held a postgraduate degree in Gynaecological Nursing and Midwifery (n = 4), obstetricians (n = 1), paediatricians (n = 1), and public health experts (n = 1)) who rated each item for their relevance on a scale of 1–3 (1 being ’not relevant’, 2 ’quite relevant’ and 3 ’most relevant’). Experts were requested to explain the ratings of 2 and how such items could be modified to obtain a rating of 3. The expert suggestions included minor adjustments in words used, so the changes were made, and all items that initially received a rating of 2 and 3 were retained. The obstetrician, paediatrician and public health expert were selected based on 1) their long track of supporting midwifery, 2) their understanding of professional midwifery gained through long-term exposure to midwife-led care models outside of India and 3) their understanding of the context of practising professional midwifery in India. In this study, slight modifications were made in all competencies, except those related to the ’pedagogy of midwifery’, ’ethical’, and ’legal’ aspects of midwifery, where major modifications were made to break down complex theoretical concepts into several specific, action-based items.

#### Step 2: Testing the accuracy of the translation and comprehensibility of the questionnaire

*Linguistic accuracy*. The questionnaire was developed in English and translated into the official languages of the six states selected for data collection: *Assamese*, *Hindi*, *Gujarati*, *Kannada and Telugu*. Translation was carried out by nurse-midwives/ public health experts native to one of these states and fluent in both English and native language. Six rounds of linguistic validity were carried out in 2018–2019 to ensure that each version of the translated tool captured the exact meaning as intended in the English tool. In the end, a dual-language questionnaire–English as the mandatory language combined with one of the five local languages spoken in the given state–was utilised to ensure better and uniform understanding among the respondents. This decision was driven by the fact that all midwifery tutors in India must have at least a bachelor’s degree in nursing, which is offered only in English, but they could still feel more comfortable reading and responding to the questionnaire in their native language.

*Face validity*. Mosier (1947) defined face validity as a process establishing that a test should appear meaningful to those taking the test [38]. The translated tool was checked for clarity, non-ambiguity and simplicity level of each item through think-aloud interviews with 20 midwifery tutors. These tutors were not included in the actual study. The tutors read each item on the self-assessment scale aloud and reflected on what they understood it to mean. A research assistant maintained detailed item-wise notes to record how clear, non-ambiguous, simple, and relevant the 20 tutors found each item. Minor changes were made in items based on respondents’ remarks to increase the clarity of the questionnaire. No items were added or deleted in any competency.

#### Step 3: Evaluating the psychometric properties of the questionnaire

*Construct validity*. Construct validity establishes the extent to which the questionnaire measures the construct it is supposed to measure [37]. Exploratory Factor Analysis is an empirical process used for newly constructed scales to 1) reduce redundant items from the questionnaire and 2) explore the dimensions of the construct that the questionnaire has captured [37]. Exploratory Factor Analyses using Principal Component Analyses with Direct Oblimin Rotation were carried out in this study.

Direct Oblimin Rotation is recommended for analysis when the construct is believed to be multi-dimensional, and the dimensions are theoretically expected to correlate [37]. The Kaiser-Meyer-Olkin (KMO) test assessed the sample adequacy for running Principal Component Analysis [37]. Bartlett’s test of Sphericity was carried out to check the patterns of variance in respondents’ responses [37]. Kaiser’s criterion of Eigenvalue ≥1 guided the process of identifying factors (hereafter called domains; equivalent to sub-scales in the scaling process) to develop the component matrices for each competency [37]. Multi-factorial loading was prevented by limiting the item coefficients to ≥0.40.

Since each competency addressed an independent aspect of educator competency, the Principal Component Analysis was run individually for each competency instead of only once for all the items. This was in line with emerging evidence that a grand total score of multidimensional scales may be inaccurate in measuring the trait/feature [39].

*Item analysis*. Item analysis calculates the strength of the relationship between an item and the nature of the content intended to be measured [37]. Items having a correlation coefficient of >0.25–<0.75 were retained in the questionnaire under relevant Principal Components.

*Subscale analysis*. Each emerging subscale extracted from the Principal Component Analyses was evaluated to check the internal correlations between 1) the total score for given competency and identified sub-scales, 2) the total score for given competency and individual item scores, and 3) the sub-scale score and individual item-scores included in the subscale.

*Internal reliability*. Cronbach’s α coefficient, which shows the degree to which a set of items are interrelated when measuring a single construct [37], was used to test internal reliability. An internal reliability score of ≥0.7 is acceptable for a newly constructed scale [37].

*Sampling methodology*. A three-step sampling methodology was applied. In the first step, the states were selected based on their Maternal Mortality Ratio (MMR) to test the tool in some states reporting high, some reporting near-national-average and some reporting low MMR. All districts in selected states that had at least one education institute with at least one cohort of midwifery students (integrated programme) were purposively selected. A state-wide pool of educational institutes offering integrated midwifery courses was developed. In the second step, educational institutes were randomly selected. In the third step, within the institutes, all midwifery tutors meeting the eligibility criteria were purposively invited to participate.

*Study sites*. The educational institutes offering midwifery education across six Indian states, Assam, Bihar, Gujarat, Karnataka, Telangana, and Uttar Pradesh (UP), were selected based on their MMR. Based on the National Family Health Survey (2017) by the Government of India, Assam and UP had an MMR of>200; Bihar and Karnataka had an MMR between 101 and 199; and Gujarat and Telangana reported an MMR of <100 [40]. In addition, the six states represented the north, northeast, west, central and south regions of India. In total, 158 districts were visited out of a total of 241 districts across six states selected for this study, based on the availability of an educational institute offering a midwifery programme.

In the six states, the probability Proportional to Size (PPS) sampling method was used to randomly select educational institutes that met the eligibility criteria for this study: 1) approved by State Nursing Councils to run nursing with integrated midwifery programmes, and 2) Well established school/college of nursing with at least one cohort of midwifery students graduated (either degree or diploma course).

In total, 526 educational institutes were visited. Based on the Indian Nursing Council’s (INC) criteria of a 1:10 tutor: student ratio for optimum teaching, a minimum of 2–6 midwifery educators were expected to be present in each of these institutes based on the number of approved student seats mentioned on the INC website in 2018.

*Sample size*. As the sample size of <250 is considered poor, 500 is considered fair, and 1000 and above is considered excellent for psychometric evaluation [37], attempts were made to recruit all midwifery tutors who met eligibility criteria pending their consent.

*Study respondents*. All tutors meeting the eligibility criteria: 1) having taught at least one cohort of midwifery students for at least one academic year; 2) currently teaching midwifery in classroom/laboratory and/or clinical set-up in an institute within selected states were invited to participate.

*Data collection*. Data collection was carried out from October 2019 to March 2020 at the selected educational institutes (n = 526) in an allocated room allowed by the institute’s Principal to interact with the midwifery tutors. The draft questionnaire was converted into an e-form using Computer Assisted Personal Interviews (CAPI) software [41]. All potential respondents at the education institutes were provided with detailed written information about the study by trained Research Assistants (RAs) with previous experience in survey methods. Midwifery tutors who signed the written informed consent form were offered e-tablets specifically carried out by RAs for the purpose of data collection. All respondents read and responded to the digital self-assessment questionnaire themselves. Due to the settings of e-questionnaire, no items could be missed if the participant wished to submit the responses. The RAs worked under the direct supervision of State Research Coordinators. The State Coordinators provided daily reports to the first and last co-authors. PIs also made regular quality checks on processes adopted by field teams for 1) selecting respondents from available groups of eligible midwifery tutors, 2) consent-taking and data collection, 3) maintaining field records and 4) completeness and quality of uploaded data using e-questionnaire. Data collection was carried out so that it did not interfere with the teaching schedule of the institutes.

*Ethical considerations*. The Ethical Review Board of the Foundation for Research in Health Systems, Bengaluru, Karnataka state, India, granted the ethical clearance to undertake this study (IRB Reg. No.: IRB0009235/2019/2). Written informed permission was obtained from the state government authorities in all six states to conduct the study, especially in the public sector institutes. While the government approvals directed all educational institutes to cooperate in the study, potential participants were informed that they could refuse participation if they wished to, without any fear of reporting and adverse consequences. Written informed consent was procured from each educator who agreed to participate. The respondents ’ names and other personal information were coded to maintain anonymity. All forms where the respondents chose to stop midway due to any reason were removed from the analyses.

### Findings

In total, 2016 midwifery tutors representing the public and private sector educational institutes (n = 526) from the six states consented to participate in this study. The average time taken to complete the questionnaire was just over 42 minutes. The study had a response rate of 90%, with the primary reason for refusal being a lack of time to participate. Nearly all respondents (96%) were women. Table 2 shows the demographic profile of the respondents.

**Table 2:** Demographic profile of participating midwifery tutors (n = 2016).

Demographic characteristic	n (%)
*Mean age in years (standard deviation)*	32.5 (9.9)
*Highest professional education (n = 2016)*	
Diploma in Nursing	51 (2.5)
B.Sc. Nursing	1280 (63.5)
M.Sc. Nursing or higher	673 (33.4)
M.Sc./Higher degree in other subjects (non-nursing/midwifery)	12 (0.6)
*Workplace (n = 2016)*	
Public sector	474 (23.5)
Private sector	1542 (76.5)
*Clinical experience in completed years (n = 2016)*	
No clinical experience before becoming an educator	863 (42.8)
<5 years experience	886 (43.9)
5–9 years experience	142 (7.0)
>9 years experience	125 (6.3)
*Total teaching experience in completed years (n = 2016)*	
< 1 year	571 (28.3)
1–5 years	983 (48.8)
6–9 years	188 (9.3)
≥10 years	274 (13.6)
*Number of midwifery programmes they were teaching currently (n = 2016)*	
Single programme	1386 (68.8)
2 programmes	448 (22.2)
3 or more programmes	182 (9.0)

#### Content validity of questionnaire

In this study, CVI scores for draft items (n = 119) ranged from 0.52 to 1. Deleting items with CVI <0.70 resulted in the removal of 31 items out of 119, and ultimately, 88 items (20, 15, 8, 10, 8, 11, 7, 5 and 4 items, respectively, over nine WHO core competencies) were retained. The respondents understood and found all the items during read-aloud sessions (face validity). Therefore, all 88 items were retained.

#### Item analysis

The 88 items on the draft questionnaire demonstrated positive and statistically significant (p<0.05) item-total coefficients ranging between 0.26 to 0.75. As all items were within the acceptable range, all 88 items were retained.

#### Construct validity

The KMO indices for nine WHO core competencies showed that the sample adequacy for carrying out Principal Component Analysis was achieved. Bartlett’s sphericity test results for nine WHO core competencies demonstrated that the dataset was appropriate for Principal Component Analysis. The results of KMO and Bartlett’s tests are presented in Table 3.

**Table 3:** Results for KMO test of sample adequacy and Bartlett’s test of sphericity.

List of nine WHO core competencies	KMO Test for Sample Adequacy	Bartlett’s test of sphericity (χ^2^)*
Competency 1: Midwifery educators create an environment that facilitates learning	0.964	18579
Competency 2: Midwifery educators create an environment for effective teaching of clinical practice of midwifery	0.957	13294
Competency 3: Midwifery educators conduct regular monitoring, evaluation and assessment of programmes and students	0.936	9367
Competency 4: Midwifery educators maintain current knowledge and skills in midwifery theory and practice in accordance with the best available evidence	0.926	13884
Competency 5: Midwifery educators participate in formulating policies and programme outcomes and in designing and implementing curricula	0.934	8884
Competency 6: Midwifery educators are effective communicators and function as advocates, change agents and leaders	0.943	13409
Competency 7: Midwifery educators incorporate and promote ethical aspects of midwifery care in teaching/learning activities by consistent role modelling	0.881	8287
Competency 8: Midwifery educators incorporate and promote legal aspects of midwifery care in teaching/learning activities by consistent role modelling	0.875	5719
Competency 9: Midwifery educators promote the use of research and use it to inform midwifery education and practice	0.822	4099

Note.

*All values significant at p <0.001

Principal Component Analysis using direct oblimin rotation yielded two-factor solutions for competencies 1, 2, 4, and 7, whereas single-factor solutions emerged for competencies 3,5, 6,8 and 9. Factor loadings for most items were sufficient (≥ 0.4).

Table 4 presents a summary of the Principal Component Analysis. The first column of Table 4 presents the list of nine WHO core competencies; column 2 reports the number of factors (domains/subscales) identified under each competency through PCA. Column 3 presents three sets of findings: the **item total analysis (column 3a)**, that is, how the score for each item retained for a specific core competency after PCA correlated to the total summative score of all items retained with it, and the range of correlation coefficients has been provided. The **Item-sub-scale analysis (column 3b)** is how the score of each item correlated to the total summative score of other items retained with it in a subscale within the core competency. The **sub-scale- total scale analysis (column 3c),** that is, how the summative score of all items within the sub-scale correlated to the total score for the summative score of all items retained for a core competency. The construct validity of each sub-scale is presented in terms of the numeric Eigenvalues (column 4a)–all greater than 1 –and the percentage of variance in data explained in the data by this subscale (column 4b), and the loading range of correlations for each item retained in the sub-scale (column 4c). The last column presents the internal consistency using Cronbach’s alpha values for each core competency. Supplementary File (S1 Text) presents the final validated questionnaire.

**Table 4:** Psychometric properties from Principal Component Analysis of self-assessment questionnaire for midwifery tutors.

1	2		Number of Items	Item Analysis			Construct validity (factor analysis)			Internal Reliability Cronbach’s a
				Item-total correlation range (3a)	Item-Subscale Correlation Range (3b)	Subscale-Total scale correlation (3c)	Eigenvalue (4a)	% Explained Variance (4b)	Loading Range (4c)	
**COMP 1 Creates an enabling environment for classroom teaching**	Factor 1	Competence in planning and pedagogy (items 1–9)	9	.58–.67	.65–.74	0.93	1.26	6.28	0.44–0.82	0.86
	Factor 2	Competence in developing psychomotor and affective traits among students* (items 10–20)	11	.63–.73	.66–.75	0.95	8.94	44.72	0.49–0.78	0.91
**COMP 2 Creates an enabling environment for clinical teaching**	Factor 3	Competence in organising clinical training for students (items 1–6)	6	.65–.70	.71–.79	0.91	1.00	6.69	0.89–0.56	0.84
	Factor 4	Competence in teaching and mentoring students at clinical site (items 7–15)	9	.63–.73	.69–.76	0.96	7.12	47.44	0.42–0.90	0.88
**COMP 3 Conducts regular assessment of students and programme**	Factor 5	Competence in conducting regular monitoring, evaluation, and assessment of programs and students	10	.64–.77	-	-	5.34	53.57	0.61–0.77	0.90
**COMP 4 Maintains evidence-based self-practice and teaching**	Factor 6	Competence in performing hands-on evidence-based clinical midwifery practice (items 2–7)	6	.77–.84	.81–.89	0.94	6.00	60.01	.77–.92	0.93
	Factor 7	Competence in incorporating the latest evidence into teaching (items 1, 8–10)	4	.62–.75	.70–.86	0.88	1.09	10.89	.43–.93	0.81
**COMP 5 Contributes to policy formation, curricula**	Factor 8	Competence in formulating policies and programme outcomes and in designing and implementing curricula	8	.75–.80	-	-	4.96	62.05	.75–.81	0.91
**COMP 6 Communicates, advocates, and leads effectively**	Factor 9	Competence in effective communication and functioning as advocates, change agents & leaders	13	.61–.75	-	-	7.02	53.98	0.62–0.79	0.93
**COMP 7 Incorporates ethical aspects**	Factor 10	Competence in demonstrating to students, the resolution of ethical dilemmas (items 1–3)	3	.74–.76	.888–.895	0.84	1.02	14.53	.87–.90	0.87
	Factor 11	Competence in incorporating ethical aspects of midwifery in teaching plan (items 4–7)	4	.79–.83	.82–.88	0.95	4.32	61.77	.69–.91	0.88
**COMP 8 Incorporates legal aspects**	Factor 12	Competence in promoting legal aspects of midwifery care in teaching/learning activities	5	.78–.87	-	-	3.522	70.45	.78–.87	0.90
**COMP 9 Incorporates research**	Factor 13	Competence in promoting the use of research and using it to inform midwifery education and practice	4	.83–.88	-	-	2.92	72.89	.83–.88	0.88

Note.

*Psychomotor traits mean dexterity and skills, and affective traits mean attitude

As seen in Table 4, the PCA identified the dimensionalities within each of the nine WHO core competencies where core competencies 1,2,4 and 7 presented a two-factor solution, and the remaining core competencies presented a single-factor solution. Based on these findings, it can be interpreted that the original list of items prepared after several rounds of content and face validation improved the draft questionnaire’s clarity, comprehensiveness, robustness and construct validity.

## Discussion

This study aimed to develop and psychometrically evaluate a self-assessment questionnaire for measuring the confidence of Indian midwifery tutors. The utility of a self-assessment questionnaire as a reliable tool for measuring competence is well established through other studies for both nursing and midwifery students [22, 23, 25, 42] and for professional nurses [43, 44]. The self-assessment questionnaire in this study was psychometrically analysed using Principal Component Analysis which is widely utilised in health sciences for psychometric validation of a questionnaire, especially to reduce the set of variables for regression analysis [45]. The psychometrically tested final midwifery educators’ self-assessment questionnaire in this study contained 88 skill statements spread across nine core competencies, as mentioned in the WHO midwifery educators’ core competencies document [2]. To the best of the authors’ knowledge, it is the most comprehensive tool currently available on midwifery tutors/educators’ confidence in competence measurement based on global standards.

The context and generalizability of our questionnaire had to be interpreted in the light of evidence from educator competency studies from other professional courses due to the negligible evidence available on previously used midwifery educators’ competency assessment questionnaires. Our questionnaire seems well-placed in terms of its context and comprehensive coverage of skills that pertain to midwifery educators’ confidence in competence. For example, the Principal Component Analysis revealed that the competency on "creating an environment that facilitates learning" in our self-assessment questionnaire had two domains: "planning and pedagogy"; and "helping students develop psychomotor and affective traits". Other qualitative and quantitative studies on nurse educators have identified these two domains as critical components of educator competencies [46–48]. Competency on "effective teaching of clinical midwifery practice" in this questionnaire comprised of the domains: "organising clinical teaching" and "teaching and mentoring in the clinical area"; both have been reported as essential in previous studies too [48, 49].

The competency labelled "maintaining current knowledge and skills in midwifery theory and practice" in our questionnaire had two domains: "ability of midwifery educators to sustain own clinical skills"; and "incorporating evidence-based care in their teaching". Both these domains are relevant for the existing midwifery education in India: the midwifery educators have no authority in the clinical environment and may not have any opportunity to keep their skills updated [50]. This concern is commonly cited in all countries having integrated midwifery education, where midwifery educators do not have the dual roles of educators and professional midwives [9, 51].

The competency in our questionnaire for "incorporating and promoting ethical aspects of midwifery" focused on how the educators identified ethical dilemmas and assisted their students to resolve these dilemmas as midwives. Previous studies, albeit on nursing educators’ competence assessment questionnaires, have also explored similar themes [41]. On the other hand, another study from Turkey reported students’ perception on their own educators’ ethics but did not explore how it translated into students’ own learning of ethics in midwifery [48]. Similarly, educator-related studies from around the world have highlighted the importance of updating midwives on legal accountability for midwifery practice [52]; mentoring for building competence and motivation [49]; comprehensive monitoring and evaluation [48, 53]; all these components have been comprehensively covered in our questionnaire.

This large multi-centric national study generated evidence of Indian midwifery tutors’ confidence in their competence in all nine core competencies deemed essential by the WHO (2). This implies that:

1. the psychometrically validated questionnaire has the potential to be used as a cost-effective rapid-assessment programmatic tool for the policy implementers to understand the midwifery tutors’ own perceptions of their confidence in competence. This is beneficial for the midwifery tutors as it makes them self-aware of their personal learning needs; at the same time, dissemination of the findings from the use of this tool can make the policymakers and programme implementers aware of the priority learning needs requiring continuous professional development.
2. The self-assessment may motivate midwifery tutors to seek out the most beneficial training to rebuild their confidence in competence, thereby having a long-term impact on planning and implementing need-based, cost-effective on-the-job training for midwifery tutors.
3. The same tool–with slight modification in each skill statement–can also be used to collect students’ feedback on their educators’ competence in teaching midwifery. The same tool may also be used as an observational tool after necessary modifications, for a comprehensive objective assessment of the midwifery educators’ competencies.
4. The tool can also serve as a monitoring tool for any quality assurance of midwifery education intervention to better understand the impact.

### Strengths and limitations

The questionnaire was developed through a rigorous process [39]. To the best of the authors’ knowledge, this questionnaire is the first comprehensive standardised tool with a programmatic value for measuring the baseline and ongoing competence of midwifery educators. The data were collected simultaneously over the six states from a large number of midwifery tutors from both the public and private sectors (n = 2016) and was a nationally representative sample. The research team comprised of Research Assistants having previous experience of data collection during the survey, and all were provided rigorous training to ensure quality and uniformity during data collection while preserving the confidentiality and rights of the respondents. The interview bias (the risk of having different explanations of the terms to respondents by the different Research Assistants) was reduced by having each technical term use a copy of definitions/explanations for any technical term (item-wise); so that they could read it out to the respondents if asked. However, the study uses self-assessment by the midwifery educators to measure their confidence in competence and there may have been a response bias (false reporting where the respondents select an option that they do not necessarily believe to be correct) [54] from at least some of the respondents fearing a backlash on reporting poor self-competence.

As a mitigation strategy, every effort was made by the Research Team to impress upon the respondents that no punitive measure would be initiated against low-scoring respondents. The length of the questionnaire was identified during the pilot testing as a key limitation for this study. To avoid the risk for mechanical ticking of the responses, short breaks were provided to respondents during the actual data collection, and refreshments were served to the respondents during the breaks.

Literature searches were carried out in the databases PubMed, CINAHL, and EBSCOhost to find validated versions of a questionnaire measuring midwifery competency to establish convergent validity (degree of correlation between the total scores of the scale being tested and an established scale testing the same concept). However, no such questionnaire could be identified. Therefore, a convergent validity check could not be performed.

While the authors engaged in an exhaustive and rigorous process to ensure uniform comprehension of the questionnaire across all six languages (five state-languages and English); it is possible that something may still have been lost in the translation process, this was mitigated by implementing the dual-language questionnaire, therefore keeping the English version as the most authentic and abiding questionnaire.

## Conclusion

The midwifery educators’ self-assessment of confidence in competence questionnaire is a psychometrically valid tool developed in India, based on global standards of midwifery educator competencies; and can be contextualised for use in other countries as well. This tool has programmatic relevance as it presents a cost-effective and sustainable solution to create self-awareness among the educators on their own competencies and to identify felt needs for seeking relevant training to improve their ability to be competent midwifery educators. The tool can be used for periodic self-assessment at the national- and state levels in the current midwifery initiative adopted by the Government of India. While, due to the risk of response bias common in self-assessment, parallel application of another objective method to monitor educator competency, such as direct observations, may be useful to further substantiate the findings in future studies, the tool will serve as a template to assess improvements and discover gaps in tutor competencies over time and before and after trainings.

## Supporting information

S1 Text Final validated self-assessment of confidence in competence questionnaire for midwifery tutors based on WHO core competencies. (DOCX)
