# Supplementary material for: Developing and validating a tool for assessing the confidence in the competence of midwifery tutors in India on WHO core competency domains
Source: PLOS Glob Public Health. 2024 Aug 29;4(8):e0003626. doi: 10.1371/journal.pgph.0003626 (PMC11361588; doi:10.1371/journal.pgph.0003626)
Supplement: S1 Text — (DOCX) [file pgph.0003626.s001.docx]

**Final Validated Self-Assessment of Confidence in Competence Questionnaire for Midwifery Tutors based on WHO Core Competencies**

**Competency 1: Midwifery educators create an environment that facilitates learning.**

**Please answer the following questions**

1. Do you ever get involved in revising/adapting/updating midwifery curriculum for your students? Yes No
2. Is there a curriculum committee in your institute? Yes No
   1. When was the committee's last meeting held? ________ months ago ___________ years ago __________Do not know
3. Based on my own assessment of my knowledge, ***in general,*** I rate my knowledge regarding **creating an environment that facilitates learning** that forms the basis of high quality, culturally relevant and appropriate care for women-newborns and childbearing families as:

*(Move the slider to rate your self-assessment of knowledge in this area on a scale of 0 to 100 OR place a cross on the line below to mark your assessment of self-knowledge on a scale of 0-100 WHERE the left end point of the line denotes 0 knowledge and the right endpoint of the line denotes 100% knowledge)*

The following table lists knowledge and skills adapted from the WHO list of tutor competencies. Please read every statement carefully and answer the questions related to your awareness and confidence for each knowledge/skill statement

|  | **Competency 1: Midwifery educators create an environment that facilitates learning.** | **Not aware of this item/not relevant** | **How confident are you in performing this item independently?**  **1: Not confident**  **2: Little confident**  **3: Somewhat confident**  **4: Quite confident**  **5: Very Confident**  **(Please mark your self-rating)** |
| --- | --- | --- | --- |
|  | **Skills** |  |  |
| A1 | I can develop my teaching sessions with knowledge of theories of learning |  |  |
| A2 | I can engage students in active learning |  |  |
|  | I can use the following teaching approaches reflecting midwifery theory and practice: |  |  |
| A3 | - problem-based-learning |  |  |
| A4 | - case studies/case narratives |  |  |
| A5 | - group discussions |  |  |
| A6 | - seminars/workshops |  |  |
| A7 | - I update the syllabus every academic year |  |  |
| A8^*^ | I can prepare a structured lesson plan for each session |  |  |
| A9^*^ | I can use appropriate AV aids (classroom; lab, clinical setup) |  |  |
| A10 | I can advocate for adequate clinical equipment made available for my students for clinical practice |  |  |
| A11 | I can plan for clinical experience to suit students' learning needs |  |  |
| A12 | I can guide my students to develop a clear cognitive understanding of concepts |  |  |
| A13 | I can guide my students in applying their knowledge to hands-on practice |  |  |
| A14 | I can inculcate correct attitude/professional behaviour among my students |  |  |
| A15 | I can facilitate their ability to practice procedures independently |  |  |
| A16 | I can treat midwifery students as adult learners |  |  |
| A17 | I can facilitate their ability to maintain polite and assertive communication with ward staff and women |  |  |
| A18 | I can inculcate in them a sense of professional pride |  |  |
| A19 | I can inculcate in them a habit of critical self-evaluation |  |  |
| A20 | I can inculcate in them the habit of self-learning |  |  |

^*^The two items did not load on any subscale during PCA. Researchers are recommended to take a discrete call to use or delete in the future use of this tool.

**Competency-2. Midwifery educators create an environment for effective clinical teaching of midwifery care**

1. Are you involved in planning clinical placements for your students? Yes No
2. Is there a designated clinical supervisor for your students at the clinical site? Yes No
3. Do you have meetings about student clinical learning/performance with the staff/professionals present at the clinical site? Yes No
4. If yes, when was the last meeting held? ___________________________________
5. Based on my own assessment of my knowledge, ***in general,*** I rate my knowledge regarding **creating an environment for effective clinical teaching of midwifery care** that forms the basis of high quality, culturally relevant and appropriate care for women-newborns and childbearing families as:

(Move the slider to rate your self-assessment of knowledge in this area on a scale of 0 to 100 OR place a cross on the line below to mark your assessment of self-knowledge on a scale of 0-100 WHERE the left end point of the line denotes 0 knowledge and the right endpoint of the line denotes 100% knowledge)

|  | **Competency-2. Midwifery educators create an environment for effective clinical teaching of midwifery care** | **Not aware of this item/not relevant** | **How confident are you in performing this item independently?**  **1: Not confident**  **2: Little confident**  **3: Somewhat confident**  **4: Quite confident**  **5: Very Confident**  **(Please mark your self-rating)** |
| --- | --- | --- | --- |
|  | **Skills** |  |  |
| B1 | I can plan clinical posting for my students |  |  |
| B2 | I can use hospital census data to determine the number of students to be posted for getting the required experience (as per INC norms) |  |  |
| B3 | I can gain cooperation from staff nurses to allow students to practice |  |  |
| B4 | I can gain cooperation from residents/physicians/ obstetricians for allowing students to practice |  |  |
| B5 | I can help the student to build trust with women |  |  |
| B6 | I can arrange for appropriate personal protective equipment for my students |  |  |
| B7 | I am capable of providing clinical supervision to students |  |  |
| B8 | I can protect the women and baby from any harm during students' practice |  |  |
| B9 | I can provide constructive criticism/remedial measures to my students on their clinical competence |  |  |
| B10 | I can delegate work among students for optimum service to women/babies/families |  |  |
| B11 | I can establish an open student-teacher relationship while maintaining professional boundaries |  |  |
| B12 | I can create a non-judgmental learning environment in my sessions |  |  |
| B13 | I can demonstrate a new skill in clinical setup before having the students practice it |  |  |
| B14 | I can demonstrate appropriate communication (respectfulness, assertiveness) |  |  |
| B15 | I can be patient with students requiring additional educational inputs |  |  |

**Competency 3: Midwifery educators are responsible for conducting regular monitoring, evaluation, and assessment of programs and students.**

1. Have you received education about the principles and methods of assessing student performance? Yes No
2. Are there guidelines for assessing student performance from INC or the state nursing council? Yes No
3. Do you have the flexibility to design assessments of student’s knowledge and skill in the classroom, laboratory, and clinical area? Yes No
4. What are the various tools used for ongoing assessment of student performance? For example, checklists for health education, casebooks, etc. Please mention them in the table below.

______________________________________

1. Based on my own assessment of my knowledge, ***in general,*** I rate my knowledge regarding **conducting regular monitoring, evaluation, assessment of programs and students.** that forms the basis of high quality, culturally-relevant and appropriate care for women-newborns and childbearing families as:

*(Move the slider to rate your self-assessment of knowledge in this area on a scale of 0 to 100 OR place a cross on the line below to mark your assessment of self-knowledge on a scale of 0-100 WHERE the left end point of line denotes 0 knowledge and the right endpoint of the line denotes 100% knowledge)*

|  | **Competency 3: Midwifery educators are responsible for conducting regular monitoring, evaluation, assessment of programs and students.** | **Not aware of this item/not relevant** | **How confident are you in performing this item independently?**  **1: Not confident**  **2: Little confident**  **3: Somewhat confident**  **4: Quite confident**  **5: Very Confident**  **(Please mark your self-rating)** |
| --- | --- | --- | --- |
| C1 | I can assess student’s learning after each session for example planning an exercise |  |  |
| C2 | I can regularly test my students' learning outputs through appropriate tests |  |  |
| C3 | I can follow INC norms for pass/fail marks for students |  |  |
| C4 | I can treat my students equally while evaluating them |  |  |
| C5 | I can use structured checklists to assess my students' performance on critical, essential and desirable steps in procedures |  |  |
| C6 | I can judge my students' proficiency in performing a procedure |  |  |
| C7 | I can adjust my teaching methods to the changing proficiency among my students |  |  |
| C8 | I can take responsibility for my students' development as professional midwives |  |  |

**Competency 4:** **Midwifery educators maintain current knowledge and skills in midwifery theory and practice based on best evidence available.**

1. Are you required to assist/attend any childbirth OR practice clinical midwifery as part of your job description? Yes No
2. Approximately when did you last attend a childbirth? (Please tick in the table below)

Less than or 1 month ago Less than or 6 months ago More than 1 year ago More than or 5 years ago

1. As per your knowledge does the Indian Nursing Council have regulatory guidelines for midwifery practice in India? Yes No
2. Have you undergone an in-depth pedagogical (teaching-related) training before becoming a midwifery tutor? Yes No
3. I fulfil the Indian Nursing Council's requirements/regulations to be a midwifery tutor? Yes No
4. Do you know about any international standards /list of essential competencies for a midwifery practice? Yes No
5. Are you aware of any national/international standards/essential list of midwifery tutor competencies? Yes No
6. Are you able to keep yourself updated about latest evidence-based practices in midwifery? Yes No
7. If yes then please mention the name of the last journal you have read for the same. _____________________________________________________
8. Are you able to attend required numbers of workshops/in-service-education to maintain my license? Yes No
9. Based on my own assessment of my knowledge, ***in general*** I rate my knowledge regarding **maintaining current knowledge and skills in midwifery theory and practice based on best evidence available** that forms the basis of high quality, culturally-relevant and appropriate care for women-newborns and childbearing families as:

*(Move the slider to rate your self-assessment of knowledge in this area on a scale of 0 to 100 OR place a cross on the line below to mark your assessment of self-knowledge on a scale of 0-100 WHERE the left end point of the line denotes 0 knowledge and the right endpoint of the line denotes 100% knowledge)*

|  | **Competency 4:** **Midwifery educators maintain current knowledge and skills in midwifery theory and practice based on best evidence available.** | **Not aware of this item/not relevant** | **How confident are you in performing this item independently?**  **1: Not confident**  **2: Little confident**  **3: Somewhat confident**  **4: Quite confident**  **5: Very Confident**  **(Please mark your self-rating)** |
| --- | --- | --- | --- |
| D1 | In general, I can still perform clinical duties alongside my teaching responsibilities |  |  |
| D2 | In general, I can provide pre-pregnancy care to women |  |  |
| D3 | In general, I can provide care to women in the antenatal period (including complications) |  |  |
| D4 | In general, I can provide care to women in the intrapartum period (including complications) |  |  |
| D5 | In general, I can provide care to women in the postpartum period (including complications) |  |  |
| D6 | In general, I can provide care to women in the Neonatal health (including complications) |  |  |
| D7 | In general, I can provide care to women in the Family planning care (including complications) |  |  |
| D8 | I can demonstrate the latest evidence-based midwifery practices during laboratory sessions |  |  |
| D9 | I can demonstrate the latest evidence-based midwifery practices during clinical sessions |  |  |
| D10 | I can adjust my personal practices in line with the latest emerging evidence |  |  |

**Competency 5: Midwifery educators participate in formulating policies and programme outcomes and in designing and implementing curricula**

1. Based on my own assessment of my knowledge, ***in general,*** I rate my knowledge regarding **formulating policies and programme outcomes and in designing and implementing curricula** that form the basis of high quality, culturally relevant and appropriate care for women-newborns and childbearing families as:

(Move the slider to rate your self-assessment of knowledge in this area on a scale of 0 to 100 OR place a cross on the line below to mark your assessment of self-knowledge on a scale of 0-100 WHERE the left end point of the line denotes 0 knowledge and the right endpoint of the line denotes 100% knowledge)

|  | **Competency 5: Midwifery educators participate in formulating policies and programme outcomes and in designing and implementing curricula** | **Not aware of this item/not relevant** | **How confident are you in performing this item independently?**  **1: Not confident**  **2: Little confident**  **3: Somewhat confident**  **4: Quite confident**  **5: Very Confident**  **(Please mark your self-rating)** |
| --- | --- | --- | --- |
| E1 | I can pre-plan the required logistics for a future session |  |  |
| E2 | I can represent midwifery education amidst a multidisciplinary team (other faculties from nursing education, and medical education, ) |  |  |
| E3 | I am capable of being part of collaborative implementation of course |  |  |
| E4 | I can apply the curriculum to assess the actual versus desirable pace of learning |  |  |
| E5 | I can identify the updation required in the curriculum implementation |  |  |
| E6 | I can adjust the learning pace to remain 'on track' by modifying teaching-learning activities |  |  |
| E7 | I am able to identify gaps in the quality of midwifery education currently provided to students |  |  |
| E8 | I can develop an alternate midwifery education implementation plan to bridge gaps |  |  |

**Competency-6: Midwifery Educators are effective communicators and function as advocates, change agents and leaders**

1. Based on my own assessment of my knowledge, ***in general,*** I rate my knowledge regarding **effective communication, advocacy, change and leadership** that forms the basis of high quality, culturally relevant and appropriate care for women-newborns and childbearing families as:

(Move the slider to rate your self-assessment of knowledge in this area on a scale of 0 to 100 OR place a cross on the line below to mark your assessment of self-knowledge on a scale of 0-100 WHERE the left end point of the line denotes 0 knowledge and the right endpoint of the line denotes 100% knowledge)

|  | **Competency-6: Midwifery Educators are effective communicators and function as advocates, change agents and leaders** | **Not aware of this item/not relevant** | **How confident are you in performing this item independently?**  **1: Not confident**  **2: Little confident**  **3: Somewhat confident**  **4: Quite confident**  **5: Very Confident**  **(Please mark your self-rating)** |
| --- | --- | --- | --- |
| F1 | In general, I can express myself clearly to my students/colleagues/clinical staff, and others |  |  |
| F2 | I can demonstrate command of written communication |  |  |
| F3 | I can demonstrate command over public speaking |  |  |
| F4 | I can demonstrate an effective health education session to my students |  |  |
| F5 | I can demonstrate sensitivity to the cultural diversity of women/families/students staff |  |  |
| F6 | I can apply my knowledge of cultural diversity to safeguard the rights of women/families/students staff |  |  |
| F7 | I can demonstrate being sensitive to the gender nuances in clinical and classroom situations |  |  |
| F8 | I can prepare an action plan for change |  |  |
| F9 | I can lead the change by setting an example |  |  |
| F10 | I can demonstrate the characteristics of a leader (integrity, vision, creativity, perseverance, etc.) |  |  |
| F11 | I can carry out advocacy for the rights of midwifery women/neonates/families/students/staff |  |  |

**Competency-7: Midwifery educators incorporate and promote ethical aspects of midwifery care in teaching/learning activities by consistent role modelling**

1. Are there formally defined ethical principles for midwifery practice in India of which you are aware? Yes No
2. Do you consciously incorporate and promote ethical principles for midwifery care in teaching/learning? Yes No
3. Do you have the necessary experience to answer students' ethics-related queries? Yes No
4. If the answer to questions 21 or 22 is **YES,** please read each statement in the table below and answer questions given
5. Based on my own assessment of my knowledge, ***in general*** I rate my knowledge regarding **incorporating and promoting ethical aspects of midwifery care in teaching/learning activities by consistent role modeling,** that forms the basis of high quality, culturally-relevant and appropriate care for women-newborns and childbearing families as:

(Move the slider to rate your self-assessment of knowledge in this area on a scale of 0 to 100 OR place a cross on the line below to mark your assessment of self-knowledge on a scale of 0-100 WHERE the left end point of line denotes 0 knowledge and the right endpoint of the line denotes 100% knowledge)

|  |  | **Not aware of this item/not relevant** | **How confident are you to perform this item independently?**  **1: Not confident**  **2: Little confident**  **3: Somewhat confident**  **4: Quite confident**  **5: Very Confident**  **(Please mark your self-rating)** |
| --- | --- | --- | --- |
|  | **I can demonstrate the following ethical principles of midwifery:** |  |  |
| G1 | - No harm to women and newborn |  |  |
| G2 | - Respect for women and families |  |  |
| G2 | - Rights of women, new-borns and their families |  |  |
| G4 | I can formulate & use case studies focusing on ethical dilemmas to use during my teaching sessions |  |  |
| G5 | I can initiate a discussion on ethical dilemmas amidst my students |  |  |
| G6 | I can identify and discuss ethical dilemmas in clinical setup |  |  |
| G7 | I can demonstrate resolution of ethical dilemma in the clinical setup |  |  |

**Competency-8:** **Midwifery educators incorporate and promote legal aspects of midwifery care in teaching/learning activities by consistent role modeling**

1. Are there formally defined legal guidelines/regulations for midwifery practice in India you are aware of? (What a midwife is allowed to do and not do) Yes No
2. Do you consciously incorporate any legal regulations of midwifery care in teaching/learning? Yes No
3. Are you aware of any international legal regulatory guidelines for midwifery practice? Yes No
4. Based on my own assessment of my knowledge, ***in general*** I rate my knowledge regarding **incorporating and promoting legal aspects of midwifery care in teaching/learning activities by consistent role modeling,** that forms the basis of high quality, culturally-relevant and appropriate care for women-newborns and childbearing families as:

(Move the slider to rate your self-assessment of knowledge in this area on a scale of 0 to 100 OR place a cross on the line below to mark your assessment of self-knowledge on a scale of 0-100 WHERE the left end point of line denotes 0 knowledge and the right endpoint of the line denotes 100% knowledge)

|  | **Competency-8:** **Midwifery educators incorporate and promote legal aspects of midwifery care in teaching/learning activities by consistent role modeling** | **Not aware of this item/not relevant** | **How confident are you to perform this item independently?**  **1: Not confident**  **2: Little confident**  **3: Somewhat confident**  **4: Quite confident**  **5: Very Confident**  **(Please mark your self-rating)** |
| --- | --- | --- | --- |
| H1 | I can incorporate the legal principles of midwifery into my teaching sessions |  |  |
| H2 | I can initiate a discussion on legal dilemmas amidst my students |  |  |
| H3 | I can identify legal dilemmas in clinical setup |  |  |
| H4 | I can demonstrate how to report a legal dilemma to appropriate persons for action |  |  |
| H5 | I can ensure my students practice midwifery within the regulations applicable in India |  |  |

**Competency-9. Midwifery researchers promote the use of research and use it to inform midwifery education and practice**

1. Are you engaged in any research project? Yes No
2. Have you published any article in your career?
3. If yes, how many? Please give the number.---------------------------
4. Based on my own assessment of my knowledge, ***in general*** I rate my knowledge regarding **incorporating and promoting legal aspects of midwifery care in teaching/learning activities by consistent role modeling,** that forms the basis of high quality, culturally-relevant and appropriate care for women-newborns and childbearing families as:

(Move the slider to rate your self-assessment of knowledge in this area on a scale of 0 to 100 OR place a cross on the line below to mark your assessment of self-knowledge on a scale of 0-100 WHERE the left end point of line denotes 0 knowledge and the right endpoint of the line denotes 100% knowledge)

|  |  | **Not aware of this item/not relevant** | **How confident are you to perform this item independently?**  **1: Not confident**  **2: Little confident**  **3: Somewhat confident**  **4: Quite confident**  **5: Very Confident**  **(Please mark your self-rating)** |
| --- | --- | --- | --- |
| I1 | I can use online resources to find research and clinical guidelines relevant to midwifery |  |  |
| I2 | I can interpret and apply the published research using qualitative and quantitative approaches |  |  |
| I3 | I can guide my students in different research methodologies |  |  |
| I4 | I can demonstrate and encourage the spirit of scientific inquiry |  |  |
